# Assessment of MMP-9 and clinical characteristics in dogs with tracheal collapse based on cough severity and fluoroscopic findings: a cross-sectional study

**DOI:** 10.1186/s12917-023-03872-1

**Published:** 2024-02-10

**Authors:** Da-Yeon Jung, Su-Min Park, Ga-Hyun Lim, Kyoung-Won Seo, Ye-In Oh, Hwa-Young Youn

**Affiliations:** 1 Laboratory of Veterinary Internal Medicine, College of Veterinary Medicine and Research Institute for Veterinary Science, Seoul National University, Seoul, 00826 Republic of Korea; 2 Department of Veterinary Internal Medicine, College of Veterinary Medicine, Kyungpook National University, Daegu, 41566 Republic of Korea

**Keywords:** Tracheal collapse, Tracheobronchomalacia, Fluoroscopy, Cough, MMP-9, IL-6, SP-A, SDC-1, Dog

## Abstract

**Background:**

Tracheal collapse (TC), a common disease in dogs, is characterized by cough; however, little is known about the serum biomarkers that can objectively evaluate the severity of cough in canine TC. Furthermore, studies elucidating the relationship of fluoroscopic characteristics with the severity of cough are lacking. Therefore, this study aimed to evaluate the relationship between cough severity and clinical characteristics, fluoroscopic images, and new serum biomarkers in canine TC.

**Results:**

Fifty-one client-owned dogs diagnosed with TC based on fluoroscopic and clinical signs were enrolled in this study and divided into three groups according to the severity of cough (grade of cough: 0, 1, and 2). Signalments, comorbidities, and fluoroscopic characteristics were compared among the groups retrospectively. The serum matrix metalloproteinase-9 (MMP-9), interleukin-6 (IL-6), surfactant protein-A (SP-A), and syndecan-1 (SDC-1) levels were measured in all groups. No significant differences in age, breed, sex, or clinical history were observed among the groups. Concomitant pharyngeal collapse increased significantly with the severity of cough (*p* = .031). Based on the fluoroscopic characteristics, the TC grade of the carinal region increased significantly and consistently with the grade of cough (*p* = .03). The serum MMP-9 level was significantly higher in the grade 2 group than that in the grade 0 group (*p* = .014). The serum IL-6 level was significantly lower in the grade 1 group than that in the grade 0 group (*p* = .020). The serum SP-A and SDC-1 levels did not differ significantly among the groups.

**Conclusions:**

The severity of cough with the progression of TC can be predicted with the fluoroscopic TC grade at the carinal region. MMP-9 may be used as an objective serum biomarker that represents cough severity to understand the pathogenesis.

**Supplementary Information:**

The online version contains supplementary material available at 10.1186/s12917-023-03872-1.

## Background

Tracheal collapse (TC) is a common disease in small-breed dogs that causes chronic cough due to the flattening of the tracheal cartilage and tracheal membrane prolapse into the lumen [1, 2]. Similar to human medicine, the term tracheobronchomalacia (TBM) has been recently used in veterinary medicine to describe the involvement of bronchomalacia resulting in bronchial collapse along with TC [2]. TC can occur due to congenital or secondary causes, and chronic inflammation or other factors can exacerbate the clinical signs [1–5]. Clinical signs commonly include chronic cough described as a goose-honking sound, increased respiratory effort, and exercise intolerance [6]. TC can be diagnosed via radiography, fluoroscopy, and tracheobronchoscopy based on the clinical signs [7], and the grading of TC is based on the percentage reduction in the luminal diameter [7, 8]. However several dogs have been diagnosed with TC without a history of cough [4] or clinical features predictive of airway collapse [9], and the symptom-free period of TC has no correlation with sex, age, or the findings on the fluoroscopic images [6]. Little is known about the relevance of cough severity and TC grade. Moreover the pathophysiology of TC and the inflammatory mediators involved in disease progression are not completely understood [2, 7]. Several biomarkers have been investigated to differentiate TC from canine respiratory disease [10]; however, studies investigating the serum biomarkers of TC or TBM in both humans and dogs are lacking. Similar to canine TBM, the definitive cause of TBM in human beings is unknown; however, half of the human patients with TBM have chronic obstructive pulmonary disease (COPD) [7, 11, 12]. COPD is a chronic inflammatory lung disease that causes irreversible airway obstruction [13, 14], and the management of the primary pathology, such as COPD, is the first step in the treatment of human TBM [12]. Although dogs do not develop the same symptoms as human COPD [15], and canine TBM differs from human TBM due to anatomical differences, chronic inflammation is one of the multifactorial causes in both canine and human TBM [7, 12, 16]. So we aimed to investigate TC cases with TBM etiology with COPD markers, considering their prevalence in human medicine. From the various serum biomarkers being studied for human COPD, we selected the factors which compose the glycocalyx of the bronchoalveolar lining cells and play an important role in inflammatory process of the airway. The selected biomarkers include matrix metalloproteinase-9 (MMP-9), interleukin-6 (IL-6), surfactant protein-A (SP-A), and syndecan-1 (SDC-1), which are secreted by various cells in the airways. The serum MMP-9, IL-6, and SP-A levels are increased in patients with COPD, whereas the SDC-1 level is decreased [14, 17–21]. To the best of our knowledge, few studies have investigated the role of these biomarkers in canine TBM. Additionally, there are no established methods to evaluate the clinical symptoms in canine TC after the diagnosis or the treatment, unlike in human TBM where clinical assessment before and after treatment is possible with the pulmonary function test, 6-minute walk test, and several standardized questionnaires [22]. The images used for diagnosis are not used for monitoring the clinical severity, and we hypothesized that if there are serum indicators that differ with cough severity in dogs with TC, those serum indicators can be used as the biomarker to objectively assess and monitor the clinical status of canine TC.

This study evaluated the serum concentrations of MMP-9, IL-6, SP-A, and SDC-1 in dogs with different cough severity at the time of clinical assessment who were previously diagnosed with TC via fluoroscopy to determine their correlation with the severity of cough. This study also evaluated whether the fluoroscopic characteristics at the time of diagnosis are correlated with the cough severity during clinical follow-up. Thus, this study aimed to (1) identify a serum indicator that objectively represents the cough severity of TC in dogs, and (2) determine whether the fluoroscopic characteristics at the time of TC diagnosis can estimate the severity of cough.

## Results

### Patient data

Fifty-one dogs with TC that met the inclusion criteria were enrolled in this study and classified into three groups according to the severity of cough. Fifteen, 18, and 18 dogs were classified as grades 0, 1, and 2, respectively. The clinical characteristics of the dogs in each group are presented in Table 1. The following breeds of dogs with TC were included: Pomeranian (*n* = 15, 29.4%), Maltese (*n* = 13, 25.5%), Toy and Miniature Poodle (*n* = 8, 15.7%), mixed (*n* = 4, 7.8%), Chihuahua (*n* = 4, 7.8%), and Shihtzu (*n* = 3, 5.9%). One dog (2.0%) of each of the following breeds was also included: Beagle, Silky Terrier, Spitz, and Yorkshire terrier. The median age of the dogs was 12 years (range, 4–17 years). Twenty-two female (43.1%; three intact females, 19 spayed females) and 29 male (56.8%; one intact male, 28 castrated males) dogs were included. No significant differences in the breed (*p* = .188), age (*p* = .459), or sex (*p* = .317) were observed among the groups.

**Table 1:** Signalments, comorbidities, medications, and clinical history of the dogs in each cough grade group

Characteristic	Grade 0 (*n* = 15)	Grade 1 (*n* = 18)	Grade 2 (*n* = 18)
Age (years) (median, range)	12 (7–17)	12 (4–16)	11.5 (6–15)
Sex, n (%)			
Male	0 (0%)	1 (5.6%)	0 (0%)
Male castrated	8 (53.3%)	8 (44.4%)	12 (66.7%)
Female	1 (6.7%)	0 (0%)	2 (11.1%)
Female spayed	6 (40%)	9 (50%)	4 (22.2%)
Breed (n)	Toy and Miniature Poodle (5)	Pomeranian (6)	Pomeranian (6)
	Pomeranian (3)	Maltese (5)	Maltese (5)
	Maltese (3)	Toy and Miniature Poodle (2)	Chihuahua (4)
	Mixed (2)	Mixed (2)	Toy and Miniature Poodle (1)
	Shihtzu (1)	Shihtzu (1)	Shihtzu (1)
	Spitz (1)	Silky terrier (1)	Yorkshire terrier (1)
		Beagle (1)	
Comorbidity, n (%)			
No comorbidity	0 (0%)	1 (5.6%)	1 (5.6%)
MMVD	10 (66.7%)	14 (77.8%)	12 (66.7%)
B1	5 (33.3%)	1 (5.6%)	1 (5.6%)
B2	3 (20%)	6 (33.3%)	4 (22.2%)
C	2 (13.3%)	7 (38.9%)	7 (38.9%)
Medication, n (%)			
None	10 (66.7%)	7 (38.9%)	3 (16.7%)
Theophylline	5 (33.3%)	11 (61.1%)	15 (83.3%)
Codeine	0 (0%)	4 (22.2%)	10 (55.6%)
≥ 3 drugs	0 (0%)	2 (11.1%)	5 (27.8%)
Clinical history, n (%)			
< 3 months	5 (33.3%)	5 (27.8%)	8 (44.4%)
3–6 months	1 (6.7%)	1 (5.6%)	0 (0%)
> 6 months	9 (60%)	12 (66.7%)	10 (55.6%)

Dogs with comorbidities accounted for 96.1% (*n* = 49), and only 3.9% (*n* = 2) of the dogs were managed for TC alone. All of the comorbid diseases were diagnosed previous to the admission, and the comorbid diseases that can induce cough included MMVD (*n* = 36, 70.6%), soft palate elongation or thickening (*n* = 20, 39.2%), and pharyngeal collapse (*n* = 21, 41.2%). The ACVIM stages of the 36 dogs with MMVD were B1 (*n* = 7, 13.7%), B2 (*n* = 13, 25.5%), and C (*n* = 16, 431.4%). The number and percentage of dogs with each comorbidity in each group are summarized in Table 1. The percentage of dogs with concomitant pharyngeal collapse increased significantly with cough severity (*p* = .031). No significant difference was observed between the concomitance of MMVD (*p* = .795), MMVD ACVIM stage (*p* = .108), and soft palate elongation or thickening (*p* = .722) among the cough groups. All dogs with comorbidities were treated or managed at the time of blood sampling.

At the time of evaluation, 39.2% (*n* = 20) of the dogs were not receiving treatment for TC, whereas 60.8% (*n* = 31) of the dogs were receiving medication for TC. The medications used included theophylline (*n* = 31, 60.8%), codeine (*n* = 14, 27.5%), montelukast (*n* = 3, 5.9%), tulobuterol patch (*n* = 3, 5.9%), bromhexine (*n* = 2, 3.9%), salbutamol (*n* = 2, 3.9%), fluticasone inhaler (*n* = 2, 3.9%), salbutamol nebulization (*n* = 1, 2.0%), and prednisolone (*n* = 1, 2.0%). All dogs under treatment for TC were receiving theophylline as the first-line drug and codeine as the second-line drug, and 13.7% (*n* = 7) of the dogs were receiving more than three medications. The number and percentage of dogs receiving each medication in the three cough groups are summarized in Table 1. The usage of drugs, including that of theophylline (*p* = .017) and codeine (*p* < .001), increased significantly as the grade of cough increased. No significant differences in the percentage of patients receiving three or more drugs were observed among the three grades of cough (*p* = .087).

The clinical history was defined as the period from the day of diagnosis to the day of clinical evaluation and blood collection. Based on the clinical history, the participants were categorized into three groups: < 3 months (*n* = 18, 35.3%), 3–6 months (*n* = 2, 3.9%), and > 6 months (*n* = 31, 60.8%). The number and percentage of dogs corresponding to each clinical history group in all cough-grade groups are summarized in Table 1. No significant relationship between the clinical history and the severity of cough was observed among the three cough-grade groups (*p* = .767).

### Fluoroscopic characteristics in all cough groups

**Fig. 1:**
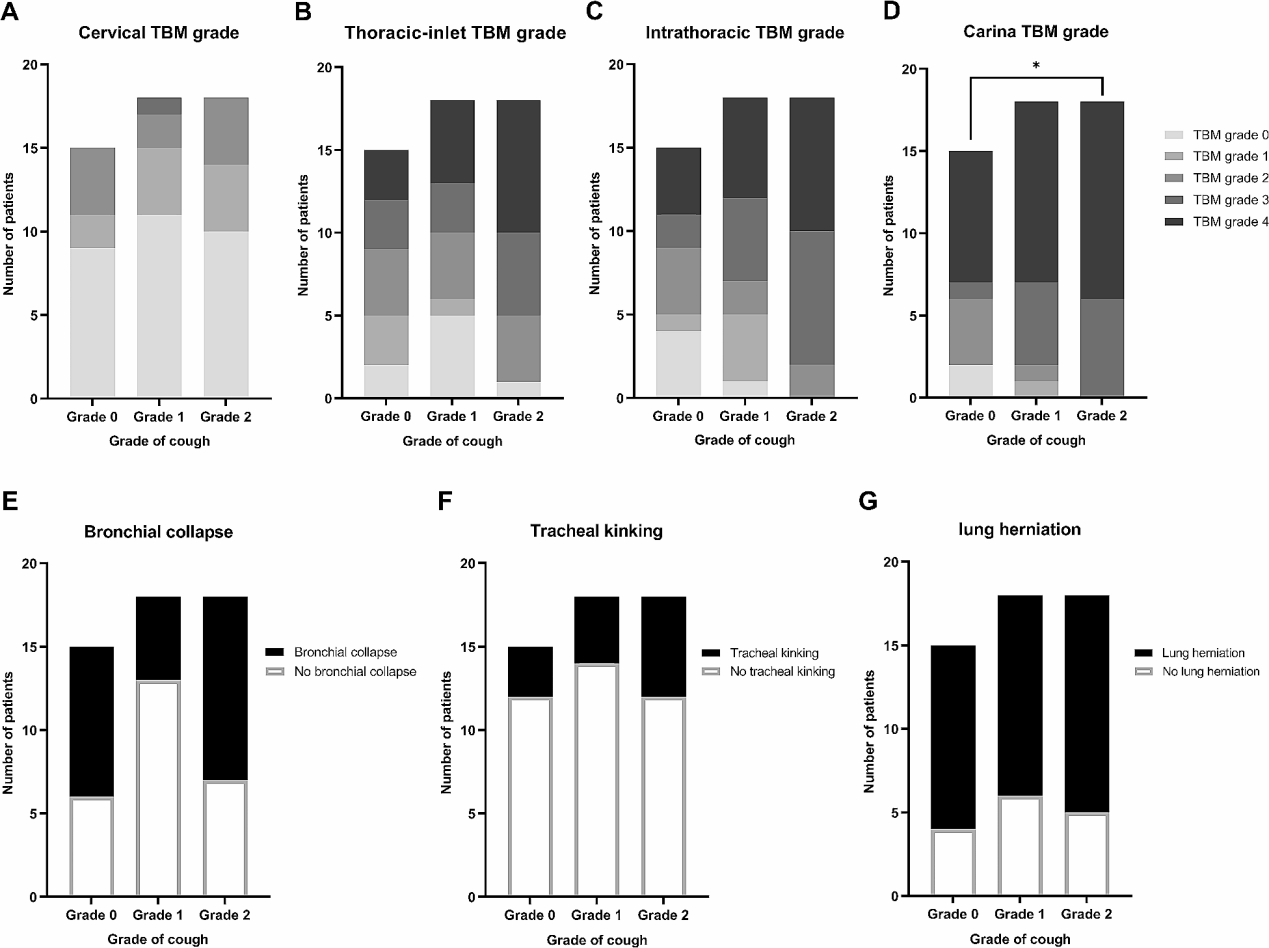
Fluoroscopic characteristics of each cough grade groups. The proportion of dogs with (**A**) cervical TBM grade, (**B**) thoracic-inlet TBM grade, (**C**) intrathoracic TBM grade, and (**D**) carinal TBM grade in each cough grade group is demonstrated. In addition, the proportion of presence or absence of (**E**) bronchial collapse, (**F**) tracheal kinking, and (**G**) lung herniation in each cough grade group is demonstrated. The grade of cough are as follows: grade 0, no cough; grade 1, < 3 times a day; and grade 2, ≥ 3 times a day or ≥ 5 min of cough at a time, or severe cough with cyanosis. The TBM grade was evaluated by radiologists based on the percentage reduction in the luminal diameter as follows: grade 1, 0–25%; grade 2, 25–50%; grade 3, 50–75%; and grade 4, > 75%. *The TBM grade of the carinal region increased significantly with the grade of cough (*p* = .03). Abbreviation: TBM, tracheobronchomalacia

Fluoroscopic images were obtained previously on the day of diagnosis for all 51 dogs, and they were divided into each cough-grade group on the day of the clinical visit. The fluoroscopic grades of the cervical, thoracic-inlet, intrathoracic, and carinal regions were evaluated in all cough groups. Among the 51 cases, 48 cases (92.3%) had TC in multiple regions. TC was detected in the thoracic-inlet (84.3%, *n* = 43), intrathoracic (90.2%, *n* = 46), and carina (96.1%, *n* = 49) regions in most cases, whereas TC was detected in the cervical region (41.2%, *n* = 21) in less than half of the cases. The percentages of each fluoroscopic TC grade between the cough-grade groups in the cervical region (*p* = .851; Fig. 1A), thoracic-inlet region (*p* = .392; Fig. 1B), and intrathoracic region (*p* = .054; Fig. 1C) did not differ significantly. In contrast, the fluoroscopic TC grade of the carinal region was increased significantly in the higher cough-grade group (*p* = .03; Fig. 1D). Among the 51 cases, bronchial collapse was observed in 49% (*n* = 25) of the dogs, lung herniation was observed in 70.6% (*n* = 36), and tracheal kinking was observed in 25.5% (*n* = 13). The presence or absence of bronchial collapse (*p* = .099, Fig. 1E), tracheal kinking (*p* = .721, Fig. 1F), and lung herniation (*p* = .931 Fig. 1G) did not differ significantly among the cough-grade groups.

### Serum levels of MMP-9, IL-6, SP-A, and SDC-1 in all cough groups

Serum concentrations of MMP-9, IL-6, SP-A and SDC-1 were measured in all cough-grade groups, and the correlation between cough severity and each serum indicators were analyzed.

**Fig. 2:**
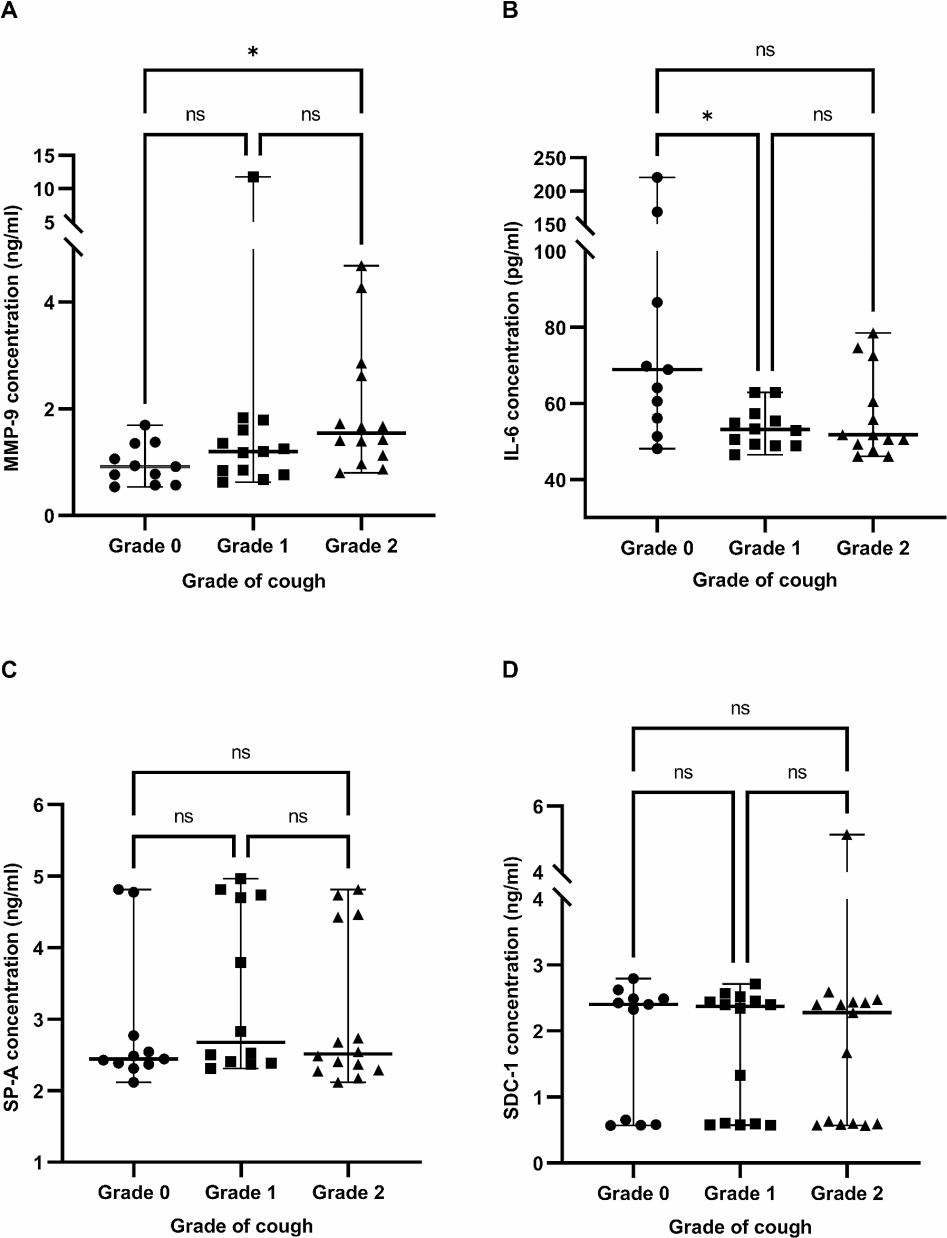
Comparison of the serum MMP-9, IL-6, SP-A, and SDC-1 levels among the cough grade groups. (**A**) The serum level of MMP-9 was significantly higher in the grade 2 group than that in the grade 0 group (*p* = .014). (**B**) The serum level of IL-6 was significantly decreased in the grade 1 group than that in the grade 0 group (*p* = .020). (**C**) The serum level of SP-A had no relationship with the grade of cough. (**D**) The serum levels of SDC- 1 had no relationship with the grade of cough. Abbreviations: MMP-9, Matrix metalloproteinase-9; IL-6, Interleukin-6; SP-A, Surfactant protein A; SDC-1, Syndecan-1

The serum MMP-9 (median, range; ng/mL) level was significantly higher in the grade 2 group (1.54, 0.80–4.69 ng/mL) than that in the grade 0 group (0.91, 0.53–1.69 ng/mL) [*p* = .014; Fig. 2A]. No significant difference was observed between the grade 1 group (1.20, 0.62–11.78 ng/mL) and other groups.

The serum IL-6 (median, range; pg/mL) level was significantly lower in the grade 1 group (53.10, 46.49–62.92 pg/mL) than that in the grade 0 group (68.93, 48.09—220.42 pg/mL) [*p* = .020; Fig. 2B]. There were no significant differences between the grade 2 group (51.70, 46.09–78.55 pg/mL) and other groups.

The serum SP-A (median, range; ng/mL) level did not differ significantly among the groups: grade 0 (2.45, 2.12–4.81 ng/mL), grade 1 (2.68, 2.31–4.97 ng/mL), and grade 2 (2.51, 2.12–4.81 ng/mL) [*p* = .46; Fig. 2C].

The serum SDC-1 (median, range; ng/mL) level did not differ significantly among the groups: grade 0 (2.40, 0.57–2.79 ng/mL), grade 1 (2.37, 0.57–2.71 ng/mL), and grade 2 (2.28, 0.57–5.14 ng/mL) [*p* = .88, Fig. 2D].

**Fig. 3:**
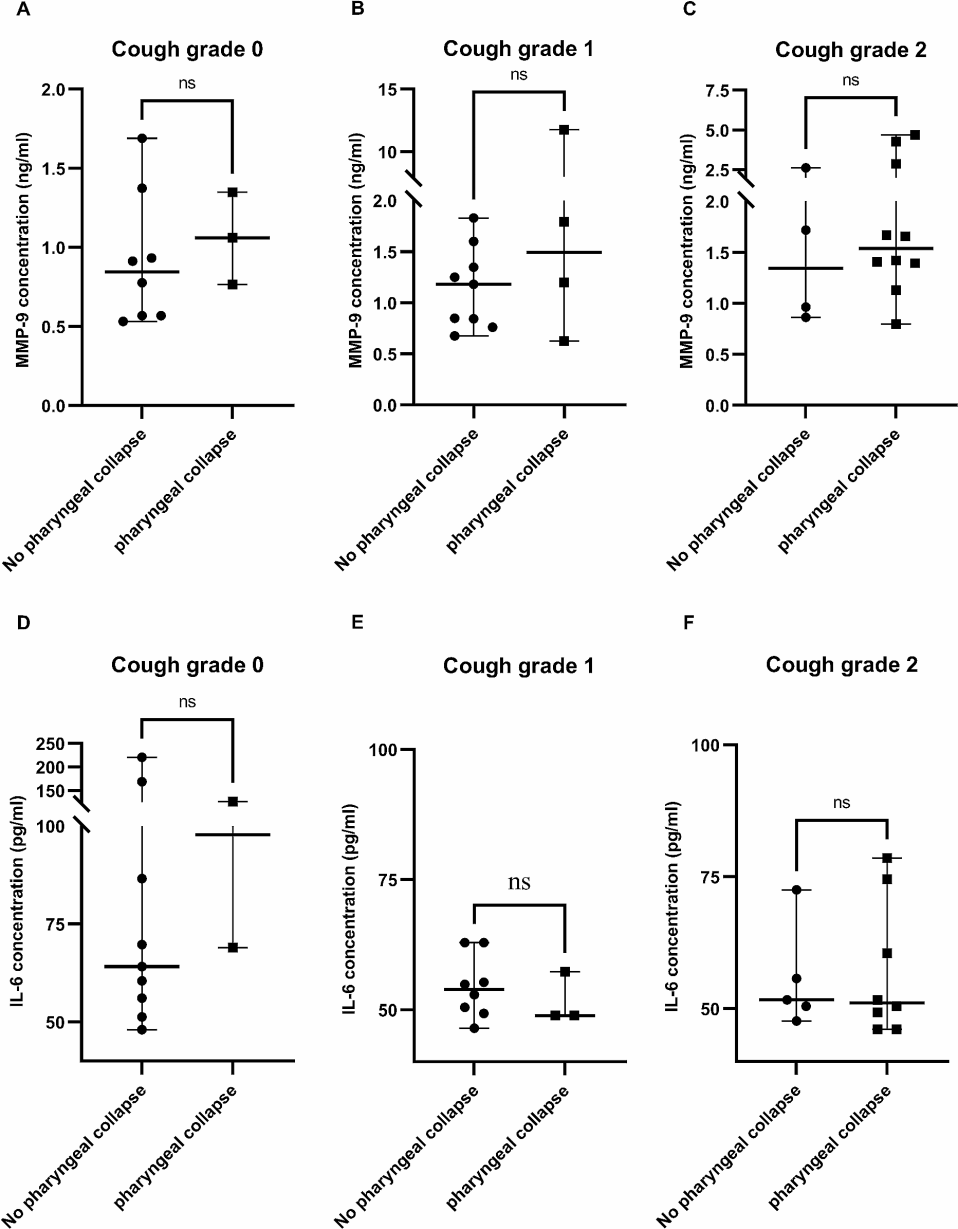
Serum MMP-9 and IL-6 levels in each cough grade groups with and without pharyngeal collapse. Regardless of the presence of pharyngeal collapse, there are no significant differences in the serum levels of MMP-9 in (**A**) the grade 0 group; (**B**) the grade 1 group, and (**C**) the grade 2 group. Similarly, regardless of the presence of pharyngeal collapse there were no significant differences in the serum levels of IL-6 in (**D**) the grade 0 group; (**E**) the grade 1 group, and (**F**) the grade 2 group. Abbreviations: MMP-9, Matrix metalloproteinase-9; IL-6, Interleukin-6

Since the concomitance of pharyngeal collapse increased significantly with the clinical severity of cough, we evaluated the relationship between pharyngeal collapse and the levels of MMP-9 and IL-6. No significant difference in the serum levels of MMP-9 was observed among the cough-grade groups, regardless of the presence of concomitant pharyngeal collapse: grade 0 (*p* = .63; Fig. 3A), grade 1 (*p* = .60; Fig. 3B), and grade 2 (*p* = .63; Fig. 3C). Similarly, no significant differences in the serum levels of IL-6 were observed within the cough-grade groups, regardless of the presence of concomitant pharyngeal collapse: grade 0 (*p* = .58; Fig. 3D), grade 1 (*p* = .47; Fig. 3E), and grade 2 (*p* = .91; Fig. 3F).

## Discussion

In the present study, we investigated the signalments, comorbidities, fluoroscopic characteristics, and serum biomarkers (MMP-9, IL-6, SP-A, and SDC-1) of 51 dogs with TC with different cough grades. Age, breed, sex, and clinical history were not related to the severity of cough. Patients with concurrent pharyngeal collapse had significantly higher grades of cough than those without pharyngeal collapse. Among the various fluoroscopic characteristics, only the TC grade of the carinal region on the day of diagnosis was related to the severity of cough at the follow-up visit. The serum MMP-9 level was positively correlated with the grade of cough, whereas the serum IL-6 level was negatively correlated with the grade of cough.

The clinical signs of canine TBM are mostly described as a harsh, dry, and honking cough, which waxes and wanes or occurs paroxysmally. Moreover, the cough is often initiated by an acute-on-chronic event [2, 7]. In human TBM, several standardized questionnaires, such as Karnofsky Performance, modified Medical Research Council (mMRC) dyspnea scale, respiratory impacted quality of life (St. George Respiratory Questionnaire), and cough specific quality of life questionnaire (CQLQ) are available for clinical assessments [22]. The cough symptom score, which consists of a two-part questionnaire (daytime and night-time symptoms), also helps score the severity of cough in humans; the scores range from 0 to 10, resulting in the total score ranging from 0 (no cough) to 10 (most severe cough) [23, 24]. However, there is no consensus regarding a questionnaire for TBM or the severity of cough in dogs; therefore, we divided the dogs enrolled in this study into the following groups based on the frequency of cough: more than three times a day, more than 5 min of cough, or a severe cough accompanied by cyanosis noticed by owners that lowers the quality of life. In human patients with TBM, the objective and subjective evaluation of symptoms is possible through pulmonary function tests and questionnaires; thus, the patient’s condition can be evaluated relatively accurately. However, forced expiratory volume, which plays a crucial role in pulmonary function tests, cannot be measured voluntarily in dogs, and there are no established methods for the objective evaluation of the clinical signs of TBM [25, 26]. Currently, the management of TBM in dogs focuses on history taking; therefore, objective indicators for evaluating the disease status are required. This study aimed to advance the management of TBM in dogs and provide a basis for understanding the etiology of TBM in the future, by investigating TC cases with TBM etiology with COPD markers, considering their prevalence in human medicine.

Similar to previous studies, most of the 51 dogs enrolled in this study were middle-aged or older small-breed dogs, and there was no sex predilection [7, 27]. Overrepresented breeds included Pomeranians, Maltese, Toy and Miniature Poodles, mixed-breed dogs, and Chihuahuas. Age, breed, and sex were found to have no relationship with the grades of cough in the present study. Moreover, no significant differences were observed in the clinical history among the cough-grade groups, indicating that the duration since the diagnosis did not affect the severity of cough on the day of clinical evaluation. The use of theophylline and codeine increased significantly with the severity of cough. All dogs under treatment for TC were receiving theophylline as the first-line drug and codeine as the second-line drug in this study. Most of the dogs with TC in the current study were middle-aged or older and had comorbidities, such as endocrine diseases, liver enzyme elevation, and chronic pancreatitis, which made it difficult to administer corticosteroid, the conventional first-line anti-inflammatory drug, repeatedly or for a long period [6]. The increased use of theophylline and codeine with the increase in the cough grade may be due to the tendency of veterinarians to prescribe these medications with the aggravation of clinical signs.

This study demonstrated that concomitant pharyngeal collapse increased significantly with the severity of cough. In a previous study, 60.7% of dogs with pharyngeal collapse had TBM as a comorbidity [28]. In the present study, pharyngeal collapse was concomitant in 41.2% of dogs with TC, suggesting that TC and pharyngeal collapse are associated. In dogs with TBM, the pressure gradient between the upper and lower airways is increased during respiration due to the narrowing of the airway, which increases resistance within the lumen [28, 29]. This altered pressure gradient imposes a chronic load and changes the tone of the dilator muscles of the pharynx, which may hinder the maintenance of the normal pharyngeal anatomy, thereby leading to pharyngeal collapse [28, 30, 31]. Cough increases the respiratory pressure gradient further [32], which may accelerate the process further. Pharyngeal collapse induces pharyngeal contraction, further worsening the cough, creating a vicious circle [33]. Dogs previously diagnosed with concurrent pharyngeal collapse had a more severe cough in this study; thus, concurrent pharyngeal collapse can be a risk factor for the progression of TBM.

The presence of concomitant MMVD had no relationship with the severity of cough. Cough is one of the main clinical signs in dogs with MMVD, which may be a result of the enlarged left atrium stimulating the cough receptors by imposing mechanical pressure on the airways [34]. A previous study reported that airway collapse was already present in all dogs with MMVD who had cough regardless of the enlargement of the left atrium, and there was no significant relationship between left atrial enlargement and the distribution of airway collapse [35]. Thus, cough in dogs with MMVD may not be related to airway collapse. Similarly, in the present study, concomitance or stages of MMVD had no significant relationship with the grade of cough in dogs with TC. This further shows that the concomitance of MMVD does not affect the severity of cough in canine TC. The concomitance of soft palate elongation and thickening had no relationship with the severity of cough. A previous study reported that concurrent soft palate elongation was observed in 7.6% of dogs with TC [36]. In contrast, in the present study, soft palate elongation or thickening was observed in 39.2% of dogs with TC.

This study also assessed the relationship between fluoroscopic characteristics and the grade of cough to determine whether the TC grade at each anatomical region and the presence of bronchial collapse, tracheal kinking, or lung herniation on the day of diagnosis are correlated with the severity of cough on the day of clinical evaluation. Among the cervical, thoracic-inlet, intrathoracic, and carinal regions, only the TC grade of the carinal region increased significantly with the severity of cough. The collapse of the cervical and thoracic-inlet regions occurs during inspiration. Similarly, the collapse of the intrathoracic and carinal regions occurs during expiration. These occur due to pressure differences within the airway during respiration [7, 27]. In previous studies, the percentage of dogs with TC presenting with collapse in each tracheal region was as follows: cervical, 16–55.3%; thoracic-inlet, 60.5–80.9%; intrathoracic, 86.9–91.5%; and carinal, 93.5– 95.7% [6, 27]. Similarly, in the present study, the percentage of collapse in the tracheal region increased sequentially from the cranial to caudal regions as follows: cervical, 41.2%; thoracic-inlet, 84.3%; intrathoracic, 90.2%; and carinal, 96.1%. A previous study reported that a history of cough was not related to TC, and many cases had fluoroscopic TC without a history of cough [4]. However, the severity of cough increased significantly with the TC grade of the carinal region in the present study. This may be related to the differences in the thicknesses of the cartilage and muscle, as the thickness of the ventral midpoint cartilage and the tracheal muscle decreases gradually from the cervical to the intrathoracic region [37]. Further studies are required to determine whether any special characteristics of the carinal region enable an easier collapse and why only the carinal region is related to the severity of cough.

Bronchial collapse, tracheal kinking, and lung herniation were found to have no relationship with the grade of cough. It is recognized that abnormalities of the cartilage of the bronchus cause bronchial collapse in dogs with TC [7, 9]. Similar to previous studies that reported bronchial collapse in 45.8– 83% of the dogs with TC, 49% of the dogs with TC had fluoroscopic bronchial collapse in the present study [7, 9, 36]. To the best of the authors’ knowledge, little is known about the relationship between bronchial collapse and cough [38]. The results of this study showed that no significant differences were observed among the groups in terms of the presence of bronchial collapse, suggesting that bronchial collapse does not worsen the cough. Another study reported that airway inflammation is not related to bronchial collapse [9]. Thus, further studies are required to identify cases in which bronchial collapse accompanies TC in dogs. Increased thoracic muscle weakness with aging and increased intrathoracic pressure may cause cervical lung herniation [4]. Previous studies have reported cervical lung herniation in 55.9–70% of all dogs that underwent fluoroscopy. A positive relationship has also been revealed between lung herniation and age, the presence of intrathoracic TC, bronchial collapse, and tracheal kinking [4, 39]. In the present study, lung herniation was observed in 70.6% of dogs with TC, and it had no relationship with the severity of cough. This is consistent with the findings of a previous study that found no association between lung herniation and chronic cough [4], suggesting that lung herniation may be the result of TC. The presence of lung herniation does not further exacerbate the clinical presentation; however, further studies are required to reveal the exact relationship between lung herniation and TC. Lastly, previous studies have reported that tracheal kinking was observed in 27–29.3% of all dogs that underwent fluoroscopy [4, 39]. In the present study, tracheal kinking was observed in 25.5% of dogs with TC and had no relationship with the grade of cough. This was in contrast with our hypothesis that tracheal kinking would aggravate the symptoms by causing damage to the trachea. In human patients with acquired TC, kinking occurs at the transition between the malacic tracheal wall and the normal segment [40]. The exact cause of tracheal kinking in dogs with TC has not been revealed; however, the weakened cartilage and increased airway resistance due to TC might be the cause. Although tracheal kinking does not manifest as cough, further studies are required on the factors that predispose dogs with TC to tracheal kinking.

A limited number of studies have investigated the serum biomarkers associated with TBM in both humans and dogs. Therefore, novel serum biomarkers of COPD that are being actively studied were used in this study [14, 17–21] to investigate TC cases with TBM etiology, considering their prevalence in human medicine and considering that inflammation exacerbates the clinical signs in both human and dog TBM. MMP-9, IL-6, SP-A, and SDC-1 were selected as the serum biomarkers, and the serum level of each factor in all cough-grade groups was evaluated. Although the levels of SP-A and SDC-1 showed no significant differences among the cough-grade groups, the MMP-9 level was significantly higher in the grade 2 group compared with that in the grade 0 group. In contrast, IL-6 was significantly decreased in the grade 1 group compared with that in the grade 0 group. As concomitant pharyngeal collapse increased significantly with the severity of cough, this study aimed to determine whether the MMP-9 and IL-6 levels were influenced by the concomitance of pharyngeal collapse. No differences in the MMP-9 and IL-6 levels were observed within each cough-grade group, regardless of concomitant pharyngeal collapse.

MMP-9, also known as 92 kDa type IV collagenase, is the predominant protease in the alveolar tissue. It has attracted attention among MMPs due to its easy detection and quantification [14, 19]. The role of MMP-9 has been studied in several canine pulmonary diseases, and the levels of MMP-9 in bronchial alveolar lavage fluid are increased in recurrent bronchopneumonia, bronchiectasis, eosinophilic bronchopneumopathy, and induced models of airway inflammation [41]. However, little is known about the serum levels of MMP-9 in respiratory diseases. There is increasing evidence suggesting that MMPs are involved in the pathogenesis of COPD [42], and recent studies have shown that MMPs and their inhibitors play a central role in lung remodeling in COPD [19, 43]. The levels of several pro-inflammatory cytokines and MMP-9 are increased during the acute inflammatory response of COPD. In addition, MMP-9 is secreted by the alveolar type II cells, alveolar macrophages, neutrophils, bronchial epithelial cells, Clara cells, endothelial cells, fibroblasts, and smooth muscle cells in the lung [14, 19]. MMP-9 degrades elastin and promotes further lung damage, and is suggested to be a key mediator in COPD [14]. The serum level of MMP-9 increases with the clinical severity and the duration of clinical history in patients with COPD [14]. This finding is similar to the result of the present study that showed a positive correlation between the serum MMP-9 level and the severity of cough in dogs with TC. This may indicate that increase of cough in TC dogs is related with elastin degradation of the airway. Further studies are required to determine whether the serum MMP-9 level is increased as a result of inflammation induced by cough or whether the increase in the pathway of inflammation induces cough.

IL-6 is a key cytokine in inflammatory storms that acts as a pro-inflammatory mediator and acute phase response inducer [18, 44]. IL-6 can be produced by different sources in the lung, such as epithelial cells, interstitial fibroblasts, macrophages, and other inflammatory cells [45]. IL-6 is produced downstream from the response to a variety of stimuli, such as allergens, respiratory viruses, exercise, environmental particles, and inhaled toxic particles [45]. IL-6 contributes to lung damage through mucus hypersecretion, matrix deposition, and protease release from granulocytes via regulatory mechanisms [44, 45]. Studies have revealed that the serum IL-6 level is elevated in patients with COPD, especially during the acute exacerbation phase [17, 44]. IL-6 antibodies have been proposed as a novel therapeutic agent for improving airflow limitation due to IL-6-induced airway mucus hypersecretion in patients with COPD [44]. In veterinary medicine, novel supplements that can alleviate inflammation and oxidative stress improved clinical signs and decreased the IL-6 and tumor necrosis factor-α (TNF-α) levels, suggesting that TC induces the synthesis and secretion of pro-inflammatory cytokines [5]. Therefore, it was hypothesized that the IL-6 levels would increase with the severity of cough; however, the serum IL-6 level was found to be negatively correlated with the severity of cough in dogs with TBM in this study, possibly due to the anti-inflammatory properties of IL-6, which inhibit TNF-α and IL-1 [18]. Decreased levels of IL-6 in cough groups may result from the decreased anti-inflammatory mechanisms, resulting in cough. This is similar to the findings of a previous study, which showed that the serum IL-6 levels were decreased in patients with idiopathic COPD compared with that of controls [46]. However, as decreased levels of IL-6 in group 2 dogs were not significant in this study, further studies with large population are required to reveal the significance, and to determine whether other factors decrease the IL-6 levels in dogs with TBM with cough compared with dogs without cough. Moreover, further studies that compare the IL-6 levels in healthy dogs and dogs with TBM would help reveal the role of IL-6 in dogs with TBM [46]. 

SP-A is a pulmonary surfactant that enhances pathogen clearance and regulates adaptive and innate immune-cell functions [47, 48]. SP-A functions as an opsonin by binding to a variety of bacteria, viruses, allergens, and apoptotic cells and is secreted by alveolar type II cells and Clara cells [47–49]. SP-A also has direct effects on immune cells, modulating the production of cytokines and inflammatory mediators [47]. At present, SP-A has been established to have a relationship with COPD in both animal and human studies and may be related to the progression and prognostic evaluation of COPD in terms of airway remodeling, inflammatory response, and clinical symptoms [20]. In a previous study, the serum SP-A levels were negatively correlated with pulmonary function tests and positively correlated with inflammatory indicators and clinical severity in patients with COPD [20]. SP-A is known to be increased when leakage occurs after the injury of basement membrane of the alveoli and vessels, or when the alveolar type II cells proliferation begins [48]. However, the present study revealed that SP-A was not related to the severity of cough in dogs with TC. This may be because the cough does not occur from the injury of alveoli and vessels in TC, and cough itself does not make the injury of the alveoli and vessels as well.

SDC-1 is the main proteoglycan of the airway epithelial cells and plays an important role in the inflammatory process [21, 50]. SDC-1 controls epithelial plasticity and promotes fibroproliferation by altering the alveolar epithelium to a profibrotic phenotype [51]. SDC-1 expression facilitates cytoprotective signals and helps limit inflammation, thereby minimizing lung injury [52]. The serum SDC-1 level is decreased in patients with COPD and has a negative correlation with lung function, exacerbation risk, and systemic inflammation. It is suggested that enhanced epithelial-mesenchymal transition process in COPD patients may have resulted in reduced expression of SDC-1 [21]. However, the present study revealed that SDC-1 was not related to the severity of cough in dogs with TC. Further studies are needed, but this may indicate that epithelial-mesenchymal transition are not enhanced with cough in dogs with TC.

This study had several limitations. First, the number of dogs enrolled in this study was limited, and no control group was included. A larger population with matched healthy controls is required to confirm the findings of the present study. Second, the fluoroscopic characteristics do not represent the case at the time of the clinical visit, as the fluoroscopic images were acquired at the time of diagnosis and not followed up. Third, most dogs were diagnosed with TC via fluoroscopy, and bronchioalveolar lavage was not performed. Lastly, most cases in this study had comorbidities and were on medications, which may have affected the serum factors, as other diseases can also induce inflammation, and medications may reduce inflammation. The MMP-9 levels can be increased in neoplastic disease and this could have affected the results. Therefore, further controlled studies on MMP-9 and IL-6 are required in the future.

In conclusion, the concomitance of pharyngeal collapse may be a risk factor for the progression of canine TC. Based on various fluoroscopic characteristics, the TC grade of the carinal region can be the major TC grade that predicts the severity of cough. With further studies, MMP-9 may be used as a new serum biomarker to objectively represent the severity of cough in canine TC. In addition, both MMP-9 and IL-6 may be used to understand the progression and management of TC. As all the serum factors used in this study are expressed by the respiratory tract cells, evaluating these markers in bronchoalveolar lavage fluid and tracheal aspirates could provide more valuable results in understanding the pathophysiology of TC with TBM etiology, when followed by further studies.

## Methods

### Case selection

Among the cases that presented to the Veterinary Medical Teaching Hospital (VMTH) between August 2022 and December 2022, 51 dogs previously diagnosed with TC based on the findings on the fluoroscopic images and the clinical signs were enrolled in this cross-sectional study. The study protocol was approved by the Institutional Animal Care and Use Committee (IACUC) of SNU (approval number: SNU-221208-3).

### Inclusion and exclusion criteria

The inclusion criteria were as follows: (1) any age and breed of dogs; (2) cases that were previously diagnosed with TC based on the fluoroscopic findings and the clinical signs observed on the day of diagnosis at VMTH of SNU; and (3) cases with cough regardless of the severity. Notably, well-managed cases with no cough at the time of the clinical visit and well-managed cases with other concomitant diseases were also included as most of the TC cases visiting VMTH of SNU did not have TC as the only diagnosis. The dogs were recieving drugs for the cormorbidities, which included hepatotonics (ursodeoxycholic acid, silymarin, zentonil, S-adenosyl-L-methionine), antibiotics (doxycycline, amoxicillin-clavulanic acid), gastrointestinal drugs (esomeprazole, omeprazole, metoclopramide, famotidine, camostat, Lypex), hormone drugs (levothyroxine, trilostane), anticonvulsants (phenobarbitial, levetiracetam, zonisamide, Kbr), immunosuppressant (prednisolone, mycopheonolate mofetil, cyclosporine, leflunomide) and heart drugs (pimobendan, furosemide, benazepril, enalapril, spironolactone, furosemide, sildenafil, torsemide).

The exclusion criteria were as follows: (1) cases with comorbidities that affected the lung parenchyma, such as pneumonia, pulmonary mass, and pulmonary edema that were not being managed on the day of blood collection. These cases were diagnosed via radiography, C-reactive protein levels, computed tomography, and the response to medication; (2) cases suspected to have TC but not diagnosed via fluoroscopy; and (3) cases in which the cough severity was unavailable on the day of the clinical visit.

### Data collection for clinical evaluation

The medical records of the dogs enrolled in this study were acquired on the day of their clinical visit. The following information was collected: signalment; severity of cough; fluoroscopy; comorbidity, including soft palate elongation or thickening, pharyngeal collapse, and myxomatous mitral valve disease (MMVD) with American College of Veterinary Internal Medicine (ACVIM) stage; and medication history. The concomitant soft palate elongation or thickening, and pharyngeal collapse were diagnosed incidentally by the radiologist using fluoroscopic images acquired on the day of TC diagnosis. Pharyngeal collapse included both partial and complete collapse. All dogs included in this study had a previous record of auscultation performed by an internalist and radiograph interpreted by a radiologist. The dogs were diagnosed with MMVD and staged as MMVD ACVIM stage B1, B2, or C by the internalist using both the radiographic and echocardiographic measurements, based on the ACVIM consensus [53]. Dogs with MMVD ACVIM stage C were included only when the disease was well-managed and pulmonary edema was not suspected by clinical signs. Radiographs were acquired on the day of blood collection.

### Diagnosis of TC

TC was diagnosed previously based on the fluoroscopic findings and the clinical signs at the time of diagnosis. These exam results were obtained retrospectively, and the clinical signs at the time of diagnosis were mainly chronic paroxysmal goose-honking cough, accompanied by exercise intolerance or respiratory distress in some cases. Fluoroscopy of the normal respiration phase and the forced expiration phase (cough phase) was performed (Supplementary Material 1), and the dogs were positioned in right lateral recumbency or sternal position if they had severe respiratory distress. For dogs without cough during fluoroscopy, the forced maneuver by cervical trachea compression was performed for the cough phase. The grade of TC by location (cervical, thoracic-inlet, intrathoracic, and carina) at the cough phase were evaluated by radiologists. TC was graded based on the percentage reduction in the luminal diameter as follows: grade 1, 0–25%; grade 2, 25–50%; grade 3, 50–75%; and grade 4, > 75% [4, 6, 7, 27, 54]. 

In addition, tracheal kinking, bronchial collapse, or lung herniation observed on the fluoroscopic images were evaluated by radiologists. The dogs without a linear shape of the trachea, showing a curved appearance in the normal respiration phase or cough phase were considered to have tracheal kinking. Bronchial collapse was diagnosed via fluoroscopy when the main bronchi were observed to have collapsed during cough phase. Lung herniation was considered to be present when the lung lobe was herniated cervical to the seventh cervical vertebra during the cough phase in the fluoroscopic acquired in humanoid position [4]. 

### Evaluation of the severity of cough

Since there are no objective methods or subjective questionnaires to evaluate the frequency or severity of cough in veterinary medicine, we created the following standard to grade the severity of cough: grade 0, no cough; grade 1, frequency less than three times a day; and grade 2, frequency more than three times a day, duration of more than 5 min at a time, or a severe cough accompanied by cyanosis. Information regarding the average frequency and severity of the cough during the previous 1–2 weeks was obtained from the owners on the day of the clinical visit, and the dogs were divided into three groups according to these grades.

### Sample collection and preparation

Blood samples were collected from the jugular or cephalic vein by a veterinarian and stored in tubes without anticoagulants (BD Vacutainer® SST™ II Advance blood collection tubes) on the day of the clinical visit. The serum was extracted by centrifugation at 4000 × g for 3 min at 4 °C within 10 min of blood collection. After the serum samples were used to run the required hematological examination to manage the concomitant diseases, the remaining serum was stored in Eppendorf tubes at -80 °C within 9 h of blood collection. The serum samples were defrosted at 37 °C as required for enzyme-linked immunosorbent assay (ELISA) analysis. The minimum storage time was 12 h and maximum storage time was 17 weeks for the serum samples.

### Measurement of the serum MMP-9, IL-6, SP-A, and SDC-1 levels

The serum MMP-9, IL-6, SP-A, and SDC-1 levels were measured using ELISA according to the manufacturer’s protocol. Canine MMP-9, SP-A, and SDC-1 ELISA kits were purchased from MyBioSource Inc. (San Diego, CA, USA). Canine IL-6 ELISA kits were purchased from RnDSystems Inc. (Minneapolis, MN). Standard dilutions for all kits were performed according to the manufacturer’s instructions, and all samples were run in duplicate. The optical density (OD) of all samples was read at 450 nm using a microplate reader, and the mean value of the duplicates was calculated.

### Statistical analysis

Statistical analyses of the data were performed using GraphPad Prism (version 9.5.0, GraphPad Inc., San Diego, CA) and SPSS (version 29.0, IBM SPSS Inc., Chicago, IL). Normality tests were performed using the Shapiro–Wilk test. Based on the results of the normality test, nonparametric tests, including the Mann–Whitney U test, Kruskal–Wallis test, and Chi-square test, were used to evaluate the differences among the groups. Dunn’s test were used as post-hoc test for the Kruskal–Wallis test. Fisher’s exact test was used instead of the chi-square test when more than 20% of the expected cell counts were less than 5. Age differences between the groups were analyzed using Kruskal–Wallis test. Breed and sex differences between the groups were analyzed using Fisher’s exact test. The Chi-square test or Fisher’s exact test was used to determine whether any differences were present between the groups in terms of comorbidities, medications, and clinical history. The Chi-square test or Fisher’s exact test was used to analyze the differences in each fluoroscopic characteristic between the groups. The Kruskal–Wallis test was used to analyze the differences in the serum levels of MMP-9, IL-6, SP-A, and SDC-1 between the groups. Pairwise comparisons between the groups of each serum indicators were analyzed using Dunn’s test. The Mann–Whitney U test was used to analyze the differences in the serum levels of MMP-9 and IL-6 between dogs with and without pharyngeal collapse. In all comparisons, *p*-values < 0.05 were considered statistically significant. All descriptive statistics for continuous variables are presented as medians (range).

### Electronic supplementary material

Below is the link to the electronic supplementary material.

Supplementary Material 1

## Data Availability

All data generated or analyzed in this study are included in the article, and any additional inquiries can be directed to the corresponding author.

## Abbreviations

TC: Tracheal collapse
TBM: Tracheobronchomalacia
COPD: Chronic obstructive pulmonary disease
MMP-9: Matrix metalloproteinase-9
IL-6: Interleukin-6
SP-A: Surfactant protein-A
SDC-1: Syndecan-1
MMVD: Myxomatous mitral valve disease
ACVIM: American College of Veterinary Internal Medicine
ELISA: Enzyme-linked immunosorbent assay
OD: Optical density
mMRC: modified Medical Research Council
CQLQ: Cough Specific Quality of Life Questionnaire
